# High Temperature Extends the Range of Size Discrimination of Nonionic Polymers by a Biological Nanopore

**DOI:** 10.1038/srep38675

**Published:** 2016-12-07

**Authors:** Fabien Piguet, Hadjer Ouldali, Françoise Discala, Marie-France Breton, Jan C. Behrends, Juan Pelta, Abdelghani Oukhaled

**Affiliations:** 1 LAMBE UMR 8587 CNRS, Cergy and Évry Universities, France; 2 LPTM UMR 8089 CNRS, Cergy University, France; 3 Laboratory for Membrane Physiology and Technology, Faculty of Medicine, Department of Physiology, University of Freiburg, Germany; 4 Freiburg Materials Research Centre, University of Freiburg, Germany; 5 Centre for Interactive Materials and Bioinspired Technologies, University of Freiburg, Germany

## Abstract

We explore the effect of temperature on the interaction of polydisperse mixtures of nonionic poly(ethylene glycol) (PEG) polymers of different average molar masses with the biological nanopore *α*-hemolysin. In contrast with what has been previously observed with various nanopores and analytes, we find that, for PEGs larger than a threshold molar mass (2000 g/mol, PEG 2000), increasing temperature increases the duration of the PEG/nanopore interaction. In the case of PEG 3400 the duration increases by up to a factor of 100 when the temperature increases from 5 °C to 45 °C. Importantly, we find that increasing temperature extends the polymer size range of application of nanopore-based single-molecule mass spectrometry (Np-SMMS)-type size discrimination. Indeed, in the case of PEG 3400, discrimination of individual molecular species of different monomer number is impossible at room temperature but is achieved when the temperature is raised to 45 °C. We interpret our observations as the consequence of a decrease of PEG solubility and a collapse of PEG molecules with higher temperatures. In addition to expanding the range of application of Np-SMMS to larger nonionic polymers, our findings highlight the crucial role of the polymer solubility for the nanopore detection.

Nanopore Resistive Pulse Sensing (Np-RPS) has promising applications in domains such as analytical chemistry, biotechnology and medical diagnostics^1^,^2^,^3^,^4^,^5^,^6^. Np-RPS is an electrical method for the label-free detection of single molecules, which upon entry into the pore cause transient blockades of its ionic conductance. Theses blockades, called resistive pulses, contain information on the molecular properties of the analyte, the most relevant parameters being their depth and their duration.

As first shown by the translocation of polynucleotides^7^, and then of synthetic polyelectrolytes^8^,^9^,^10^, through the biological nanopore *α*-hemolysin, charged polymers are electrophoretically translocated through the pore in a stretched conformation, allowing the chemical nature of the repeat units to be interrogated in sequence. This finding inaugurated the idea of using nanopores to sequence DNA. In this case, blockade durations depend on the polymer linear length and charge and on the magnitude of the electric field^7^, while the depth of block varies in accordance with the molecular size and chemical properties of each monomers^11^,^12^.

In contrast, in the case of the interaction of nonionic poly(ethylene glycol) (PEG) polymers with *α*-hemolysin at high salt concentration (KCl 4 M), it was shown that blockade duration depends non-monotonically on PEG molar mass^13^. Blockade duration first increases with PEG molar mass going from 600 to 3000 g/mol, reaches a maximum and then decreases for larger PEGs (4000 to 8000 g/mol). Coincidentally, the nanopore relative residual conductance during PEG-induced current blockades was shown to decrease with PEG molar mass going from 600 to 3000 g/mol, and to saturate for larger PEGs. This was interpreted as a result of PEGs > 3000 g/mol becoming too large to be entirely admitted into the pore, resulting in an entropic spring effect reducing the blockade duration and a saturation of the level of current blockade as the number of monomers in the pore and contributing to the current blockade reaches a maximum^13^.

In a seminal publication in 2007, Robertson *et al*.^14^ first demonstrated, using refined data analysis, that histograms of the residual conductance of *α*-hemolysin in presence of polydisperse PEG 1500 showed periodic maxima that could be related to the size of individual PEG species present in the polydisperse mixture, giving rise to the concept of nanopore-based single molecule mass spectrometry (Np-SMMS), as it was originally named. However, this term may be somewhat misleading, as this technique does not discriminate molecules on the basis of their mass, but rather by their size, as mentioned in ref. 15. Thus we prefer to use the term “nanopore-based single-molecule size discrimination” (Np-SMSD) in what follows. While this experiment has been amply reproduced also in higher throughput formats^16^,^17^,^18^,^19^,^20^, an important limitation is that size-dependence of blockade depth is lost when PEG polymers become larger than about 3000 g/mol^13^.

It seems evident that the single-monomer resolution of size-discrimination observed with the PEG/*α*-hemolysin system, and recently also with PEG/metallic-clusters/*α*-hemolysin^15^,^21^, with PEG/aerolysin^20^ and with short oligonucleotides/aerolysin^22^, can only occur if each monomer contributes to the current blockade, i.e. when the polymer is fully accommodated in the pore. In the Np-SMSD experiment, therefore, the polymer is detected *en bloc* rather than as a sequence of monomers. This requires that the dimensions of the polymer’s conformation are similar to the inner dimensions of the pore. Thus, polymer conformation becomes of prime importance in polymer/pore interaction and in the sensing resolution of the system.

The conformation in aqueous solution of nonionic polymers in general and of PEG in particular is known to depend primarily on the factors affecting solvent quality^16^,^23^,^24^,^25^,^26^. Of these, salt concentration is known to be of prime importance for polymer-pore interaction^13^ and has been studied systematically by Krasilnikov and coworkers^16^, who first showed that the frequency and duration of PEG-induced current blockades through *α*-hemolysin increased with KCl electrolyte concentration, concomitant with a decrease of PEG solubility. However, no systematic effort has been made regarding the effect of temperature. The only study mentioning the effect of temperature^27^ showed that, in the particular case of monodisperse PEG of molar mass 1294 g/mol interacting with *α*-hemolysin, local heating of the nanopore volume lead to a decrease of both duration and amplitude of the PEG-induced blocks. The decrease of blockade duration was interpreted as a decrease of the number of cations bound to PEG, while the decrease of blockade amplitude was interpreted as the unfolding of PEG, in contradiction with the known collapse of PEG upon heating^24^. The present study was therefore undertaken to study the kinetics of PEG/*α*-hemolysin interaction over a large range of PEG sizes and temperatures.

## Results

One of the main features of the *α*-hemolysin nanopore is its stability over a wide range of experimental conditions, including aqueous solution of different salts^28^,^29^ at widely different concentrations^16^,^30^, denaturing agents^31^,^32^, the variation of pH^33^,^34^ or of temperature^35^,^36^. In particular, single-channel currents through *α*-hemolysin have previously been recorded at temperature varying between 2 °C^35^ and 93 °C^36^.

**Figure 1:**
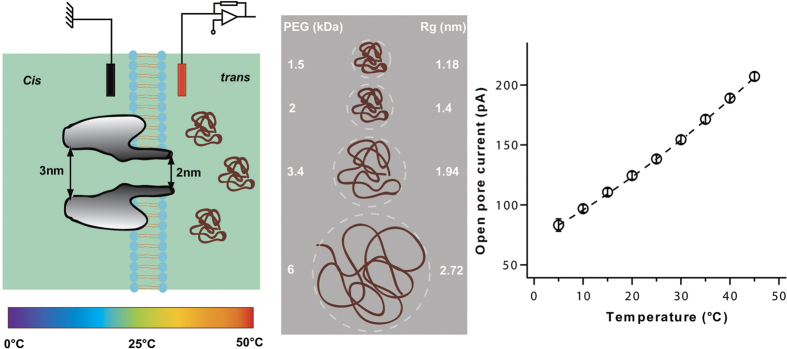
(Left) Schematic of the experimental set-up used to probe the interaction of PEGs of different molar masses with the *α*-hemolysin nanopore at different temperatures. (Center) Different molar masses of PEG used and their corresponding calculated sizes. *R*_*g*_ is the gyration radius of a coiled chain calculated assuming good solvent conditions^53^ and by considering the PEG monomer size equal to 0.35 nm^54^. (Right) Open pore current at +50 mV *versus* temperature. Data points are average values of more than 10 experiments with different temperature sequences (hot-to-cold or cold-to-hot) in order to exclude any evaporation effect. Error bars are of the same size or smaller than the symbols. The dashed line follows the variation of the buffer bulk conductance with temperature (2%/°C, see text).

A schematic of the experimental configuration is presented in Fig. 1 (left). A single *α*-hemolysin nanopore was inserted from the *cis* side of the lipid membrane. The PEG molecules were added on the *trans* side of the membrane to interact through the stem part of the nanopore. In order to obtain a robust interaction of PEG molecules with the *α*-hemolysin nanopore and to allow direct comparison with previous work^27^, all the experiments were performed in aqueous 3 M KCl solution at pH = 7.5. As both the frequency and the duration of PEG/*α*-hemolysin interactions have been shown to depend on the transmembrane voltage^16^,^28^,^37^,^38^, all the experiments were performed at a constant *trans*-positive voltage of +50 mV which corresponds to the maximal duration of PEG/*α*-hemolysin interaction under our experimental conditions^16^. The PEG/*α*-hemolysin interaction as a function of temperature was probed with polydisperse PEG mixtures of different average molar masses (1250, 1500, 2000, 3400, 6000 and 8000 g/mol) (Fig. 1 center). Assuming good solvent conditions, the calculated radius of gyration of the smallest PEG we used (PEG 1250) is approximately 1 nm (in agreement with experimental values^39^) similar to the radius of the stem of the *α*-hemolysin pore, while the radius of gyration of the largest PEG (PEG 8000) is more than 3 times larger. The temperature of the system was varied between 5 °C and 45 °C. The ionic current through a single *α*-hemolysin channel as a function of temperature is presented in Fig. 1. The pore conductance exhibits a slightly non-linear increase with temperature (≈2%/°C, see Fig. 1), following the change of the buffer bulk conductance with temperature^40^,^41^. This strongly suggests that the pore structure and dimensions are not affected by the temperature change. For all experiments, we ascertained that the variation of the pore conductance with temperature did not deviate from the calibration curve presented in Fig. 1 to avoid effects that might results from an evaporation of the buffer solution.

**Figure 2:**
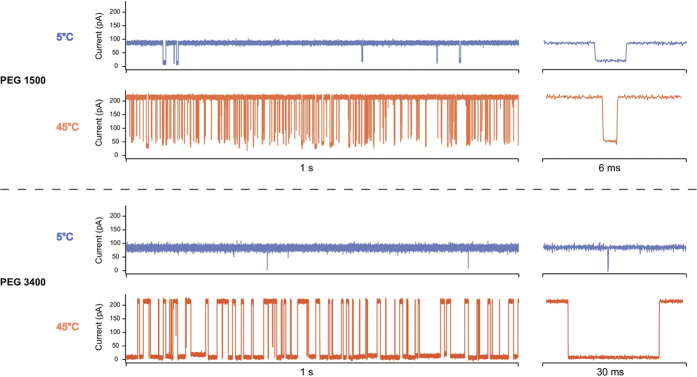
Single-channel current traces of the *α*-hemolysin pore recorded at 5 °C (blue) and 45 °C (orange) at +50 mV in 3 M KCl buffer, in presence of PEG 1500 (up) and PEG 3400 (down) added from the *trans* side of the bilayer. For each trace a typical blockade event is shown on the right.

Typical single-channel current traces of the *α*-hemolysin nanopore are presented in Fig. 2 for two polydisperse PEG mixtures of different average molar mass (PEG 1500 and PEG 3400) and two temperatures (5 °C and 45 °C). For both PEG mixtures, increasing the temperature increased the frequency of the PEG/pore interactions (blockade frequency). Surprisingly, however, the effect of temperature on the duration of the PEG/pore interactions (blockade duration) was opposite for the two mixtures. Increasing the temperature from 5 °C to 45 °C decreased the mean blockade duration by a factor of 2 in the case of PEG 1500, in qualitative agreement with what has been observed for PEG 1294 in ref. 27, while it dramatically increased the mean blockade duration by a factor of 100 in the case of PEG 3400 (see typical blockade events on the right of Fig. 2). In the case of PEG 3400, the mean blockade duration increased from ≈200 *μ*s at 5 °C to ≈20 ms at 45 °C. The mean relative residual conductance 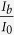, where *I*_*b*_ and *I*_0_ are, respectively, the mean blockade current and the mean open pore current, increased with temperature in the case of PEG 1500 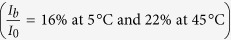, in agreement with ref. 27, while it decreased very slightly if at all 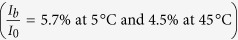 in the case of PEG 3400 (see Supplementary Fig. S2).

**Figure 3:**
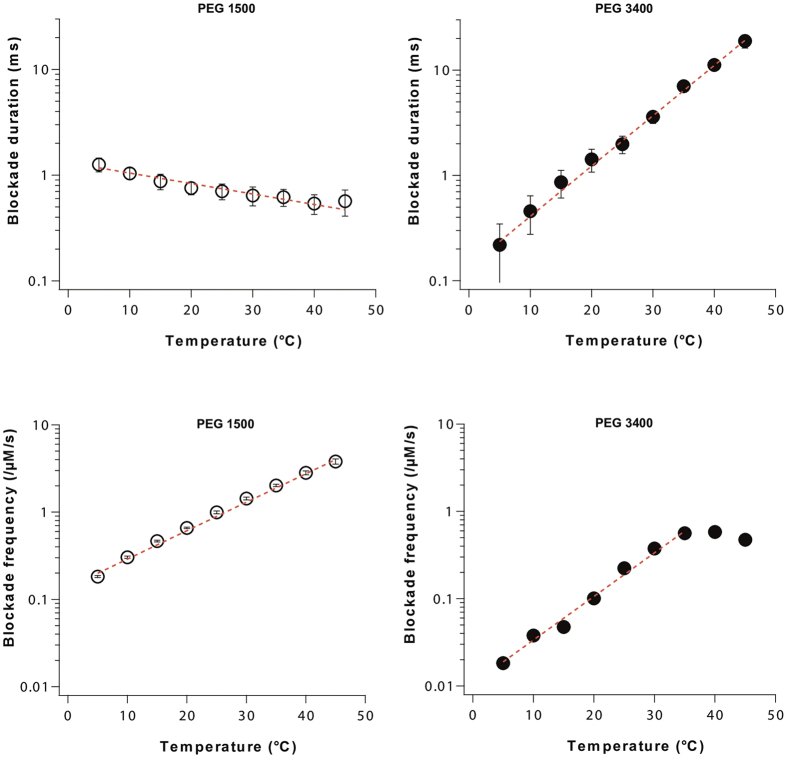
Comparison of the kinetics of interaction of PEG 1500 (left) and PEG 3400 (right) with the *α*-hemolysin nanopore as a function of temperature at +50 mV in 3 M KCl buffer. (Top) Blockade duration *versus* temperature. Dashed lines are exponential fits. (Down) Blockade frequency *versus* temperature. Dashed lines are exponential fits. For each temperature, the data point corresponds to the mean blockade duration (top) or to the mean blockade frequency (down) estimated from a single exponential fit of the blockade duration distribution and of the inter-blockade duration distribution respectively. Error bars correspond to the uncertainty of the fitting values (see the Methods section and Supplementary Fig. S1).

The blockade frequency and duration as a function of temperature are presented in Fig. 3 for PEG 1500 and PEG 3400. On the one hand, blockade frequency increases exponentially with temperature for the two PEG molar masses, saturating above 35 °C for PEG 3400 because the pore is mostly occupied due to the increasing duration of blocks. A frequency increase with temperature has previously been observed with various pores and analytes^42^,^43^,^44^,^45^ and is, here, to be expected from the lower solution viscosity if entry of PEG molecules into the pore is limiting for the on-rate of the interaction^27^. However, like Reiner *et al*.^27^, we note that, while the open pore current exhibited a 2.5 fold/40 °C increase between 5 °C and 45 °C (see Fig. 1), the blockade frequency showed a 20 fold/40 °C increase for PEG 1500 and a 30 fold/30 °C increase for PEG 3400. Thus, it is unlikely that the increase in frequency is entirely due to reduced solution viscosity and other effects, which induce sensitivity to polymer size, will have to be considered. On the other hand, mean blockade duration is shown to decrease exponentially with temperature in the case of PEG 1500, in agreement with the classical behavior observed in previous work^27^,^42^,^43^,^44^,^45^,^46^,^47^,^48^, while increasing exponentially in the case of PEG 3400 (Fig. 3).

**Figure 4:**
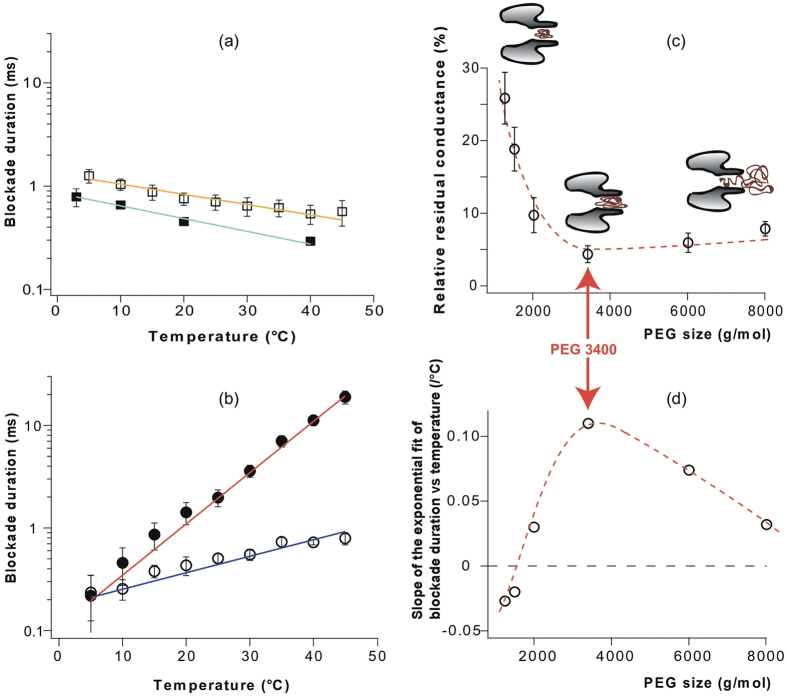
Blockade duration and relative residual conductance for PEG mixtures of different average molar masses. (**a**) Blockade duration *versus* temperature for small PEGs (PEG 1250: solid symbols, PEG 1500: empty symbols). Solid lines are exponential fits. (**b**) Blockade duration *versus* temperature for large PEGs (PEG 2000: empty symbols, PEG 3400: solid symbols). Solid lines are exponential fits. In (**a**) and (**b**), for each temperature, the data point corresponds to the mean blockade duration estimated from a single exponential fit of the blockade duration distribution and the error bar corresponds to the uncertainty of the fitting value (see the Methods section and Supplementary Fig. S1). (**c**) Relative residual conductance 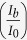 *versus* PEG molar masses. Values represent the mean of all temperatures (5, 10, 15, 20, 25, 30, 35, and 45 °C). Error bars represent the range of experimental values when the temperature was varied between 5 °C and 45 °C. Cartoons illustrate hypothetical configuration of a PEG molecule interacting with the pore depending on PEG size^13^. (**d**) Slope of the exponential fit of blockade duration *versus* temperature (represented in (**a**) and (**b**)) as a function of PEG molar mass. Dashed lines in (**c**) and (**d**) are guides for the eye.

In order to explore this new phenomenon, we systematically measured the variation of mean blockade duration with temperature for polydisperse PEG mixtures of different average molar masses between PEG 1250 and PEG 8000. As shown in Fig. 4(a), PEG 1250- and PEG 1500-induced blockade duration decreased moderately with temperature (~2 fold/40 °C) while the temperature-dependent increase in blockade durations illustrated for the larger PEGs in Fig. 4(b) was 4 fold/40 °C for PEG 2000 and 100 fold/40 °C for PEG 3400. For all PEG mixtures tested, the slope of the exponential fit to the variation of blockade duration with temperature is reported in Fig. 4(d) as a function of the average PEG molar mass. A negative slope signifies a decrease of blockade durations when the temperature increases and *vice versa* and the magnitude of the slope represents the strength of the temperature effect. While, as shown before, heating decreases the blockade duration for PEGs shorter than PEG 2000 (PEG 1250 and 1500), mean blockade durations were increased for all longer PEGs (PEG 2000, 3400, 6000, and 8000). Interestingly, the steepness of the increase of blockade duration with temperature reaches a maximum for PEG 3400, which also causes the deepest ionic current blockade in the hemolysin pore regardless of the temperature (Fig. 4(c) and Supplementary Fig. 3). Indeed, the mean relative residual conductance due to the block by a PEG molecule of the pore rapidly decreases with molar mass for PEGs shorter than PEG 3400, reaches a minimum for PEG 3400, and then slightly increases again for longer PEGs (Fig. 4(c)). This is in line with the idea that PEG 3400 corresponds to the largest PEG that fits the inner volume of the hemolysin stem under our experimental conditions (see cartoons in Fig. 4(c)).

**Figure 5:**
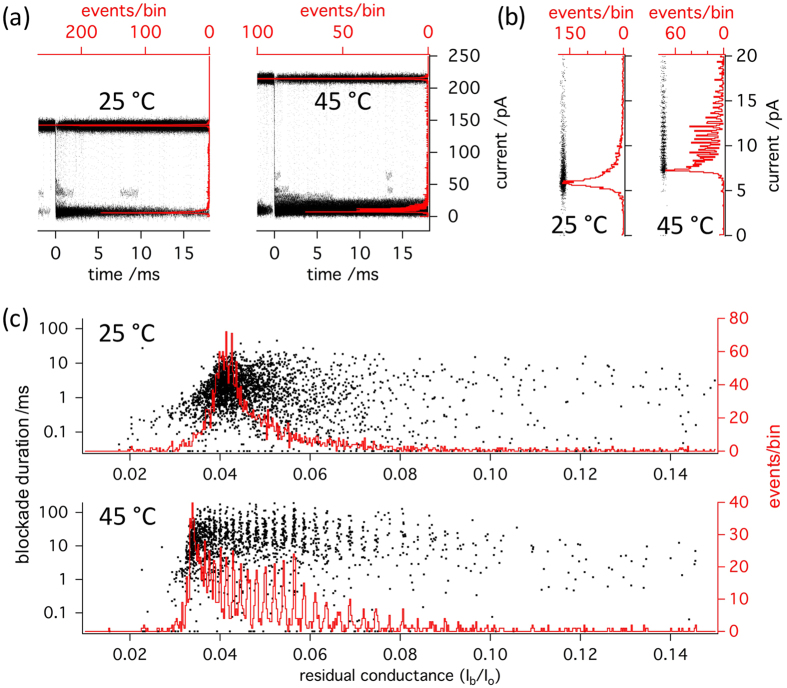
Temperature dependence of the distribution of current levels during interaction of PEG 3400 with *α*-hemolysin. (**a**) Black traces: superimposed current (right axis) *versus* time (bottom axis) plots of individual blockade events at 25 °C (n = 104) and 45 °C (n = 112). Events are aligned at the midpoint of their onset (t = 0). For each event, the last 2 ms of current *versus* time recording before the blockade event, during which another blockade sometimes occurred, are shown before before t = 0. Note the prolongation of the blockade durations with temperature from 25 to 45 °C. Red traces: histograms of mean current values of all blocked and open levels detected. (**b**) Density plots (black dots) and histograms (red traces) of blocked state current values. Note the appearance of preferred amplitudes with increasing temperature from 25 °C to 45 °C. (**c**) Histograms of relative residual conductance 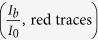 with superimposed scatterplots of blockade duration *versus* 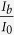. At 45 °C, approximately 20 individual PEG species can be discerned from the maxima, while no maxima are visible at 25 °C.

**Figure 6:**
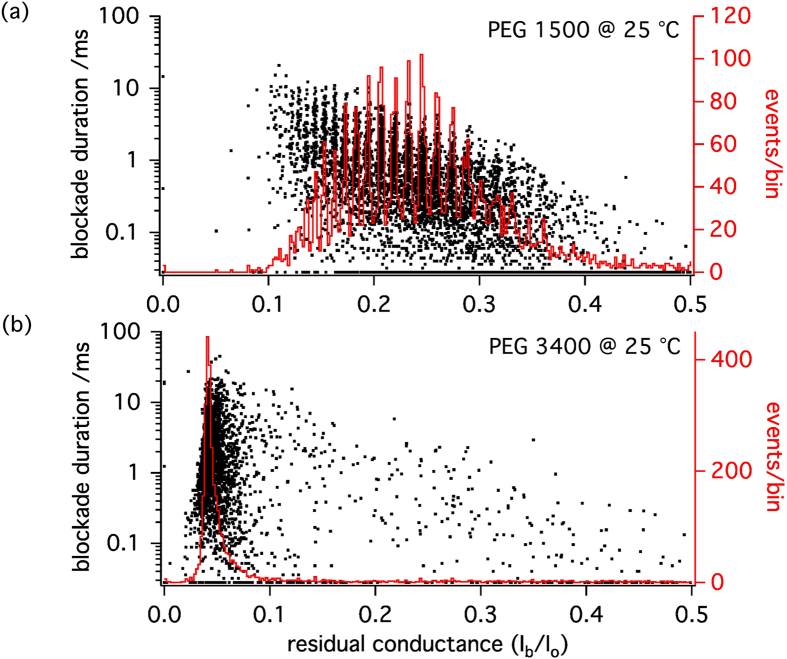
Scatter plots of blockade duration versus 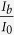 (black dots) and superimposed histograms of 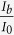 (red traces) for PEG 1500 (**a**) and PEG 3400 (**b**) at 25 °C. Even though the mean blockade duration is 3 times shorter in the case of PEG 1500, single-monomer resolution at 25 °C is achieved for PEG 1500 but not for PEG 3400 at this temperature.

The increasing temperature sensitivity of blockade durations for PEGs > PEG 1500 may suggest that higher temperatures allow them to interact more strongly with the pore. In fact, the analysis in Fig. 4 suggests that the previously observed decrease of dwell times for PEG mixtures exceeding a particular mean molar mass^13^ can be reversed by heating, restoring a positive relationship between mass and blockage durations. We therefore attempted to ascertain whether the mass-sensitivity of the blockade depth, which is equally known to be lost with PEGs surpassing a certain size^13^, would also be restored. In order to do so, we performed a detailed analysis of the temperature-dependence of the distribution of blocked pore current levels in the presence of the polydisperse PEG 3400 mixture. Figure 5(a) shows superimposed traces of blockade events aligned at their onset (time 0) for 25 and 45 °C. It can be appreciated that with increasing temperature, the open pore current increases vigorously, while the closed pore current shows only very little increase. It is for this reason that the parameter 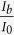 declines on average, suggesting a deeper block of the pore. However, as also shown by the histograms of the event-averaged current levels superimposed on the traces, the distribution of current levels widens considerably upon heating from 25 °C to 45 °C. A close-up of these histograms in Fig. 5 clearly discloses that this widening of the distribution is accompanied by the appearance of preferred current amplitudes at 45 °C. This is not due to binning artifacts, as shown by the abrupt appearance of periodic density fluctuations in the un-binned point plots (insets) on moving from 25 °C to 45 °C. Finally, histograms of the relative residual conductance 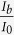 and scatter-plots of blockade duration as a function of 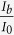 are presented in Fig. 5(c) for 25 °C and 45 °C. Clearly discernable maxima, each of which corresponding to a given polymer length^14^,^17^,^20^, are present only in the 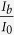 histogram obtained at 45 °C allowing to distinguish between more than 20 different PEG species with single-monomer resolution. From the superimposed scatter plots of blockade durations (dwell times), as well as from the superimposed traces it can be appreciated that the appearance of the periodic maxima is associated with an increase in the dwell times of the individual blockade events. However, the events recorded at 25 °C are well resolved (see Fig. 5(a)) and preferred amplitudes would have been detected if they had been present. Therefore, we conclude that the increase of the size-discrimination resolution with temperature is not due to the increase of blockade duration. Indeed at 25 °C, while PEG 1500 blockade duration is shorter than for PEG 3400 (Fig. 3 (up)), single-monomer resolution is achieved for PEG 1500 but not for PEG 3400 (Fig. 6).

## Discussion

Our interpretation of the temperature effect on the PEG blockade amplitude, frequency, duration and on the PEG size discrimination is largely based on the temperature effect on PEG conformation and its consequence on the PEG/pore interaction.

Regarding the effect of temperature, it has long been known that PEG is a stimuli-responsive polymer in the sense that its aqueous solutions have an upper temperature limit of solubility known as lower critical solution temperature (LCST) above which hydrogen-bond-mediated hydration of its ether oxygens is thermally compromised^24^,^26^. Thus, in contrast with biological macromolecules such as proteins, for which heating induces the unfolding of the molecule, increasing temperature leads to PEG collapse by coil-to-globule transition inducing a decrease in molecular dimensions^23^,^24^,^25^. Preceding precipitation, coil-to-globule transition, or polymer collapse, consists in a continuous reduction in the physical dimension of the polymers^49^,^50^ which, however, remain in solution. In long polymers, collapsed globular domains may coexist with stretches of random coil. Importantly, larger polymers exhibit a lower LCST. For example, 2 mM of PEG 5000 precipitates at 135 °C in water while 2 mM of PEG 30000 precipitates at 115 °C^24^. Furthermore, the LCST also depends on the solvent quality. It seems possible that, under our high salt conditions and in the highly confined space of the nanopore, the LCST of PEGs is lowered and that the temperature variations in the range we explore greatly affect the PEGs conformation.

We can therefore interpret the observed effects of temperature as follows: upon heating, PEGs tend to undergo a coil-to-globule transition. This speeds up diffusion and diminishes the entropic cost of partitioning in the pore, thus increasing blockade frequency regardless of the PEG molar mass. However the increase of blockade frequency is much stronger for larger PEGs because there is a larger entropic barrier for their entry in the nanopore, and also because they have a smaller LCST. Following Krasilnikov *et al*.^13^, we interpret the increase or decrease (depending on the PEG molar mass) of the blockade duration with temperature in relation to the number of PEG monomers that can be accommodated in the nanopore. For large PEGs (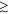
PEG 3400), at room temperature, a length of the polymer chain cannot be accommodated in the pore once it has been filled and acts as an entropic spring. This part of the chain, in order to exert such an effect, must be mobile outside the pore, hence must exist in a random coil state. Upon heating, more of the large polymers enter a collapsed state, favorising their partition into the pore. In this situation, the blockade durations increase as more of the monomers are able to interact with the pore wall and the entropic pull is reduced. This effect is much more important than thermal agitation, and is maximal in the case of PEG 3400 for which all the monomers can be accommodated in the pore and the entropic pull is removed when the temperature increases. The mean relative residual conductance remains quasi-constant upon heating in the case of PEG 3400 as the additional monomers entering the nanopore compensate the reduction of the molecular dimensions due to polymer collapse. Consistent with this picture, at higher temperatures (≥35 °C), monomeric size sensitivity for the larger PEGs is achieved as every monomer contributes to the current blockade. For short PEGs, increasing the temperature increases the relative residual conductance due to polymer collapse, as already observed in the case of single elastin-like polypeptide loops attached inside the *α*-hemolysin nanopore^51^. The increase of PEG/pore interaction with temperature is weaker than in the case of larger PEGs. Thus the blockade durations increase only modestly with temperature for PEG 2000 or even decrease for shorter PEGs for which the thermal agitation dominates. Note that an existing model of PEG/*α*-hemolysin interaction^37^,^52^, while predicting correctly the decrease of the blockade durations with temperature for small PEGs (monodisperse PEG 1294) as the consequence of reduced cation binding, could not account for the increase of the relative residual conductance^27^.

Our interpretation is supported by experiments performed at 2 M KCl with PEG 2000, for which the blockade duration is independent of temperature while it increases with temperature at 3 M KCl (see Supplementary Fig. S5), suggesting the importance of solvent quality. Experiments performed at 3 M KCl with PEG added from the *cis* side of the membrane (vestibule side of the *α*-hemolysin nanopore) revealed an increase of the PEG blockade duration with temperature above PEG 2000 but with less intensity than in the case of PEG added from the *trans* side of the membrane (see Supplementary Fig. S6) for the case of PEG 3400), because the confinement is stronger on the *trans* side.

In summary we demonstrate that, in contrast to what has been previously observed with other analytes, increasing temperature dramatically increases the duration of the interaction between nonionic PEG polymers and the nanopore of *α*-hemolysin above a threshold in PEG molar mass (PEG 2000). This effect reaches a maximum for PEG 3400, which corresponds to the PEG causing the deepest ionic current blockade in the nanopore. Morevover we demonstrate that increasing temperature extends the size range of PEG Np-SMSD, single-monomer resolution being achieved at 45 °C for PEG 3400. We interpret our observations as a consequence of PEG collapse upon heating, which in the case of large PEGs increases the number of PEG monomers that can enter the pore, bind it, and contribute individually to the current blockade. The maximal temperature effect is observed for PEG 3400, which is the largest PEG that fits the inner volume of the *α*-hemolysin stem. We expect our findings to significantly increase the range and resolution of the detection of single nonionic large polymers in Np-SMSD experiments, and to take a crucial step towards a better understanding of the mechanism of the polymer/pore interaction. Further experiments will be performed with other molecules and other nanopores in order to check whether the temperature effect described in this article is intrinsic to the PEG/*α*-hemolysin system or if it is more universal.

## Methods

### Nanopore recordings

A classical vertical lipid bilayer setup (Warner Instruments) was used for all the experiments. The lipid membrane was formed by painting and thinning a film of diphytanoyl-phosphocholine in decane over a 150 *μ*m wide aperture separating the *cis* and *trans* compartments. Both compartments were filled with a 3 M KCl solution buffered with 5 mM HEPES and set to pH = 7.5. Two Ag/AgCl electrodes were used to apply a transmembrane voltage and to measure the ionic current. *α*-hemolysin was added from the *cis* side of the membrane. After the insertion of a single nanopore, PEG molecules were added on the *trans* side of the membrane. Different PEG concentrations were used depending on their average molar mass: 50 *μ*M PEG 1250, 50 *μ*M PEG 1500, 50 *μ*M PEG 2000, 120 *μ*M PEG 3400, 40 *μ*M PEG 6000, 100 *μ*M PEG 8000. All the PEG concentrations correspond to dilute conditions, far from the PEG overlap concentration. A Peltier device controlled by a bipolar temperature controller (CL-100, Warner Instruments) allowed to set the temperature of the solution *via* a water circuit (LCS-1, Warner Instruments) around the cell chambers. Single-channel ionic currents were recorded by an Axopatch 200B patch-clamp amplifier (Axon Instruments) in the whole-cell mode with a CV-203BU headstage. The signal was filtered using a low-pass 8-pole Bessel filter at a cut-off frequency of 10 kHz. Data were acquired at 250 kHz with the DigiData 1440A AD-converter controlled by Clampex software (Axon Instruments).

### Data treatment

Our data treatment is based on a statistical analysis of a least 2000 blockade events for each set of parameters. Considering the current distribution of a current trace, a first threshold *th*_1_ = *I*_0_ − 4*σ* is used to define every possible blockade event, where *I*_0_ and *σ* are respectively the mean open pore current and its standard deviation. A possible blockade event begins when the pore current decreases below *th*_1_ and ends when the pore current increases above *th*_1_. If the mean current value during the event is inferior to a second threshold *th*_2_ = *I*_0_ − 5*σ*, then the event is considered as a blockade event. The mean blockade frequency and duration are then estimated from a single exponential fit of the inter-blockade time distribution and blockade duration distribution respectively (see Supplementary Fig. S1). For each data point, the error bar of mean blockade frequency and duration corresponds to the largest value among: the precision of the detection and acquisition system, the standard deviation of the fit coefficient from the data analysis software, and the difference between the mean values obtained from a single exponential fit of the distribution and of the cumulative distribution (see Supplementary Fig. S1). For each polydisperse PEG mixture, the results are qualitatively reproduced by at least three independent experiments. For size discrimination with monomer resolution, the procedure detailed in ref. 17 was used. While quantitative differences might appear for the blockade frequency and duration between to independent experiments using the same PEGs, the dependence of blockade frequency and duration on temperature are quantitatively reproduced. The results presented in this article correspond to individual experiments.

## Additional Information

**How to cite this article**: Piguet, F. *et al*. High Temperature Extends the Range of Size Discrimination of Nonionic Polymers by a Biological Nanopore. *Sci. Rep.* **6**, 38675; doi: 10.1038/srep38675 (2016).

**Publisher's note:** Springer Nature remains neutral with regard to jurisdictional claims in published maps and institutional affiliations.

## Supplementary Material

Supplementary Information
