# Supplementary material for: High Temperature Extends the Range of Size Discrimination of Nonionic Polymers by a Biological Nanopore
Source: Sci Rep. 2016 Dec 7;6:38675. doi: 10.1038/srep38675 (PMC5141499; doi:10.1038/srep38675)
Supplement: Supplementary Information [file srep38675-s1.pdf]

High Temperature Extends the Range of Size Discrimination of Nonionic Polymers by a Biological Nanopore

Fabien Piguet[1,2,*], **Hadjer Ouldali**[1], **Françoise Discala**[1], **Marie-France Breton**[1], **Jan C. Behrends**[3,4,5,*], **Juan Pelta**[1], **and Abdelghani Oukhaled**[1,*]

[1]LAMBE UMR 8587 CNRS, Cergy and Évry Universities, France
[2]LPTM UMR 8089 CNRS, Cergy University, France
[3]Laboratory for Membrane Physiology and Technology, Faculty of Medicine, Department of Physiology, University of Freiburg, Germany
[4]Freiburg Materials Research Centre, University of Freiburg, Germany
[5]Centre for Interactive Materials and Bioinspired Technologies, Freiburg, Germany
[*]Corresponding authors: fabien.piguet@u-cergy.fr, jan.behrends@physiologie.uni-freiburg.de, abdelghani.oukhaled@u-cergy.fr

Supplementary Information for Publication

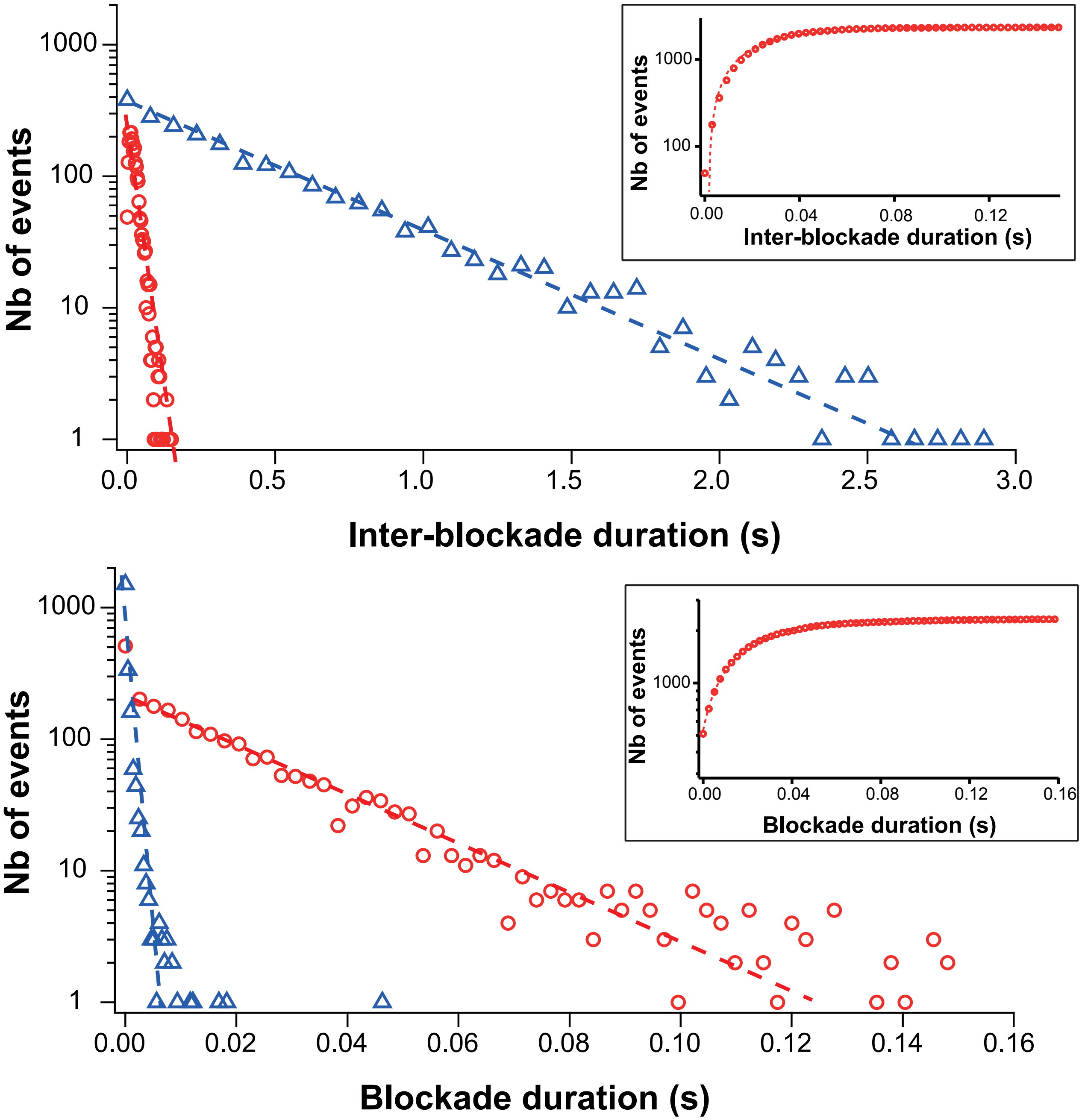

Figure 1. (up) Histograms of the inter-blockade durations in the case of PEG 3400 at 5°C (blue triangles) and 45°C (red circles). Insert shows the cumulative histogram of the inter-blockade durations at 45°C. Dashed lines are single exponential fits. (down) Histograms of the blockade durations in the case of PEG 3400 at 5°C (blue triangles) and 45°C (red circles). Insert shows the cumulative histogram of the blockade durations at 45°C. Dashed lines are single exponential fits. In the manuscript, for each data point, the error bar of mean blockade frequency and duration corresponds to the largest value among: the precision of the detection and acquisition system, the standard deviation of the fit coefficient, and the difference between the mean values obtained from the fit of the distribution and of the cumulative distribution.

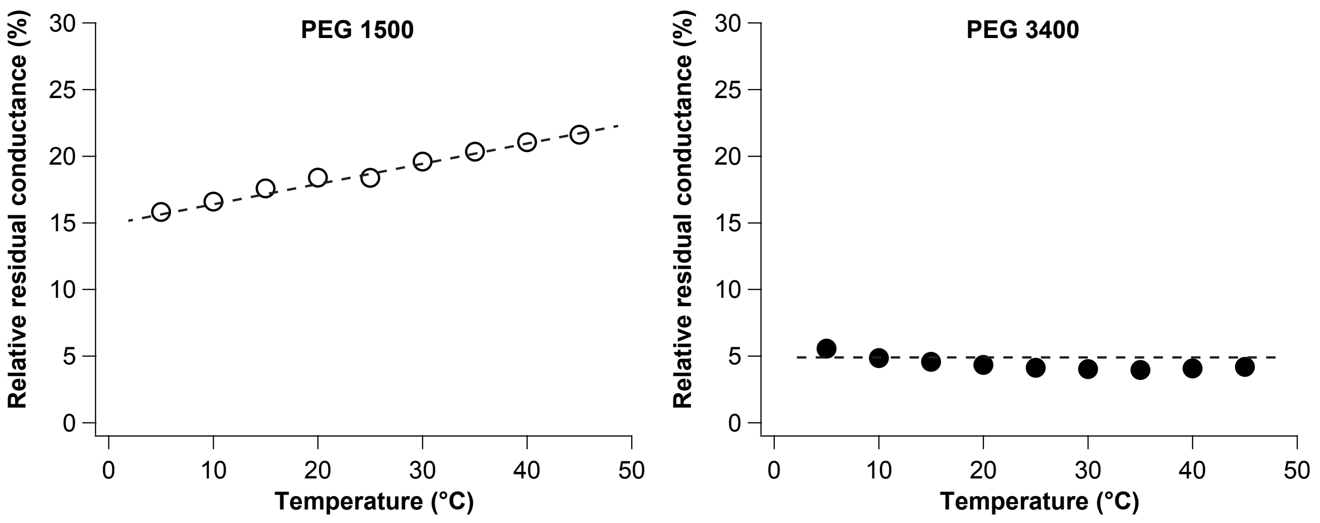

Figure 2. Relative residual conductance as a function of temperature in the case of PEG 1500 (left) and PEG 3400 (right).

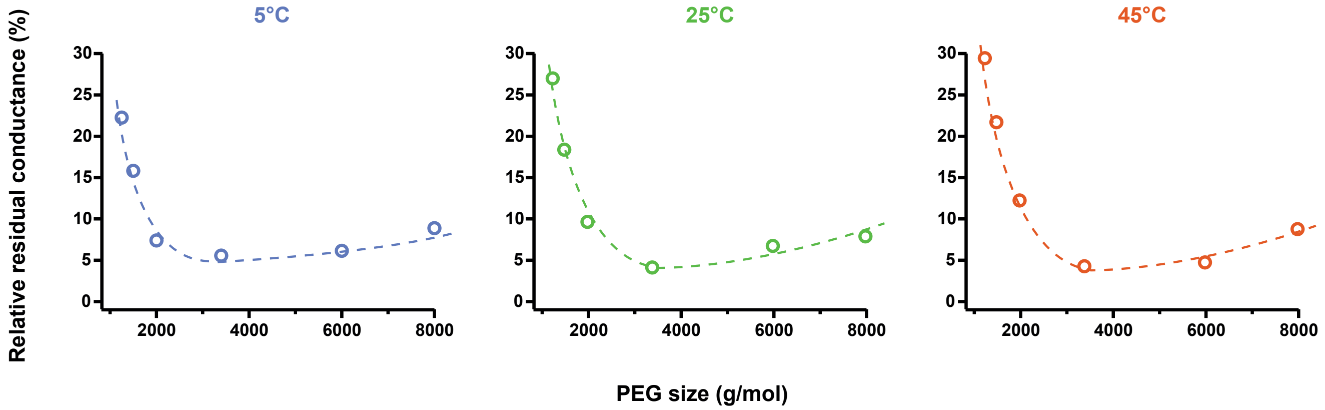

Figure 3. Relative residual conductance as a function of PEG average molar mass at 5°C (left), 25°C (center) and 45°C (right). Dashed lines are guides to the eye. Regardless of the temperature, the minimum of relative residual conductance is observed in the case of PEG 3400.

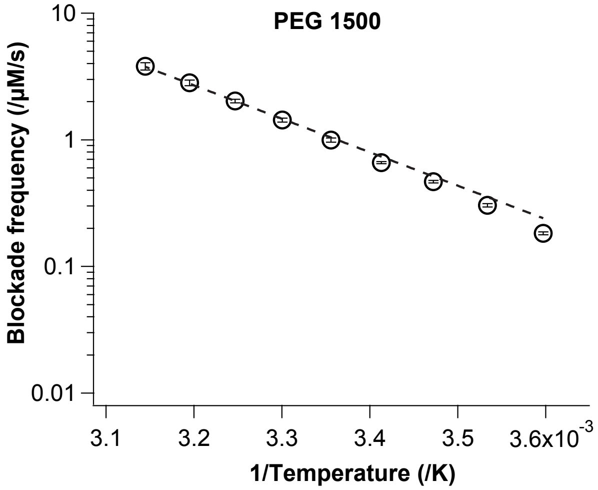

Figure 4. Determination of the entry barrier ΔF in the case of PEG 1500 (left) and PEG 3400 (right). Dashed lines are exponential fits of the form $f \sim \exp \frac{-\Delta F}{k_B T}$, leading to $\Delta F = 0.8 \times 10^{-19}$ J $\approx 18 - 21 k_B T$ for PEG 1500 and $\Delta F = 1.5 \times 10^{-19}$ J $\approx 35 - 39 k_B T$ for PEG 3400.

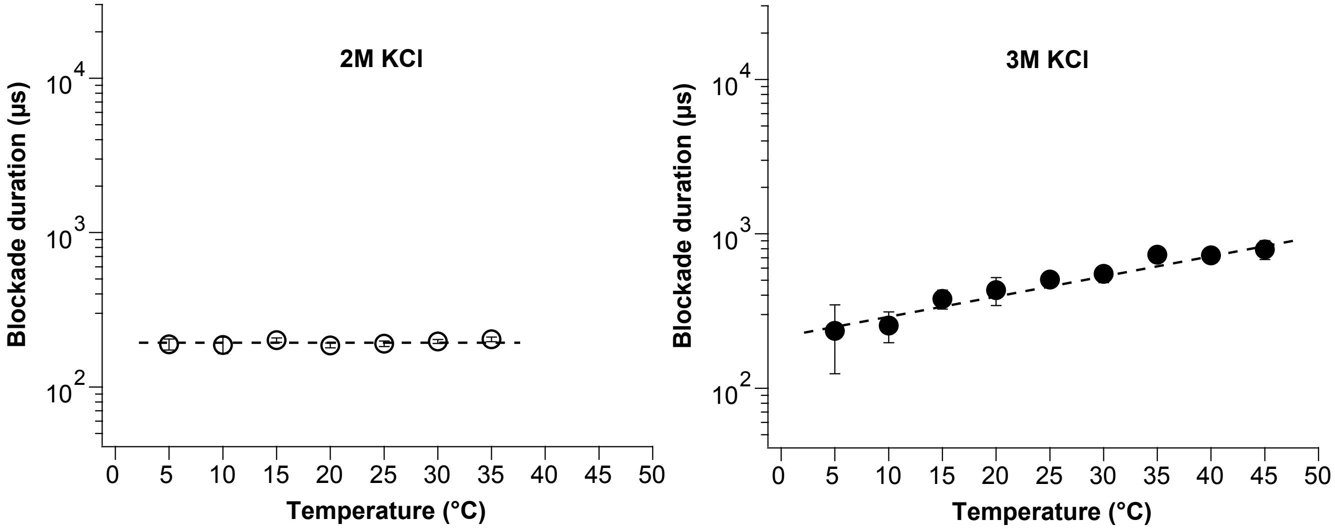

Figure 5. Blockade duration as a function of temperature for PEG 2000 in 2M KCl (left) and in 3M KCl (right).

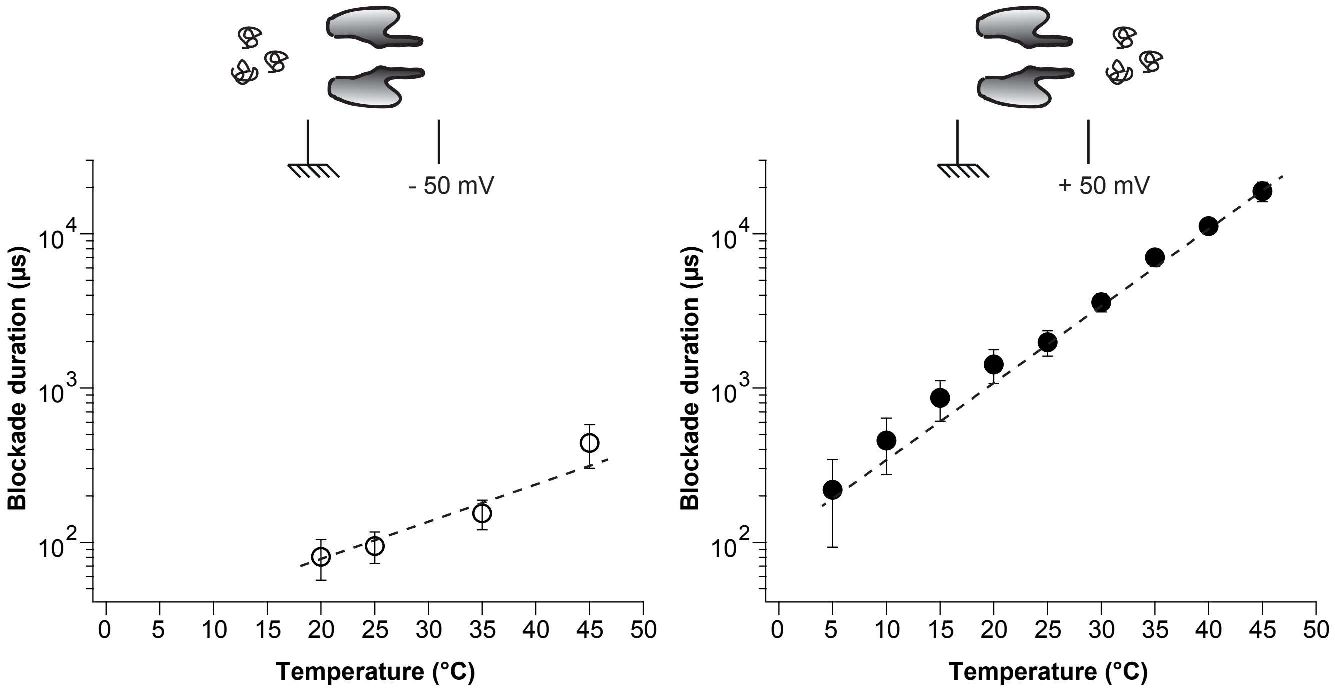

Figure 6. Blockade duration as a function of temperature for PEG 3400 added from the *cis* side (left) and from the *trans* side (right). As PEGs behave like polycations under our experimental conditions, the voltage polarity is reversed between the two experiments.
